# Open Communication and Discussion Facilitate Reconciliation: The Promise Fulfilled

**DOI:** 10.5041/RMMJ.10307

**Published:** 2017-07-31

**Authors:** Shraga Blazer

**Affiliations:** Editor-in-Chief, Rambam Maimonides Medical Journal

Dear Friends and Colleagues,

As I shared in my January 2015 editorial,^1^ the furor surrounding publication in *The Lancet* of the open letter by P. Manduca et al.^2^ carried potential for bad and for good. Here at Rambam Health Care Campus, we have chosen and will continue to look for the good.

For the benefit of those unfamiliar with the topic, I’d like to summarize the chain of events. The letter that was published accused Israel of terrible atrocities. However, there was no evidence for the accusations, and it came to light following publication that some of the authors were known anti-Semites with connections to a modern-day Nazi supporter. A storm of criticism fell on *The Lancet* for accusations leveled at Israel and its medical professionals.

At this point, critics of the Manduca et al. letter had two choices before them: (1) The path of immediate condemnation and judgement (e.g. by boycotting *The Lancet*; demanding that Elsevier dismiss the Editor-in-Chief; and pressing for retraction of the letter); (2) The path of academic discourse for the benefit of the greater good.

It is a sad commentary on the state of the academic community worldwide that only one person responded bravely and against the tide, by writing a cordial letter of invitation to Editor-in-Chief Richard Horton and inviting him to visit Israel and see the situation there for himself. That person was a Canadian immigrant to Israel, an eminent world-renowned molecular medicine and genetic researcher, the Director of Medical Research and Development at Rambam Health Care Campus in Haifa, Israel—Professor Karl Skorecki.

When people learned of his action, Skorecki received dozens of communications attacking him personally and questioning his motivations.

However, on the other side of the world, Professor Richard Horton was reading that invitation in amazement and shock—it was the only hopeful response he had received from amongst the hundreds of angry communications directed at him. In fact, despite his numerous visits to the Middle East and Israel, it was the first invitation he had ever received from Israel for the purpose of congenial academic discourse, providing him with an opportunity to meet with the men and women who had provided medical care during Operation Protective Edge and who care for the diverse population of Israel on a daily basis.

Horton’s brave decision to accept Skorecki’s invitation, with the full support of Professor Rafi Beyar, Rambam’s Director, and to enter the “lion’s den” in Israel, so to speak, has had a far-reaching and positive impact on both Israel and *The Lancet*, and hopefully beyond.

During his visit to Israel, Horton promised to launch a series in *The Lancet* on “Health in Israel” describing various aspects of Israel’s healthcare system. He stated this publicly, and it was reported in the news, albeit skeptically, worldwide.

Horton’s Rambam Grand Rounds Lecture, during which he made that announcement, was videotaped and published in *Rambam Maimonides Medical Journal* in January of 2015.^3^ As a result, I received dozens of letters from leaders in medicine around the world accusing me of being naïve, having been misled, and advising that nothing would be a good enough response unless the letter by Manduca et al. was retracted; many still called for boycotting *The Lancet* and the firing of Horton from his position as Editor-in-Chief.

Resolving conflict through academic discourse is no simple task and takes time. Many people asked, “what is happening,” “where are the results,” and “why did you allow Horton to … without any consequences?” The strongest response that we received were repeated questions regarding the lack of an apology from Richard Horton, this despite his published statements to that effect.^4^,^5^

**Figure 1:**
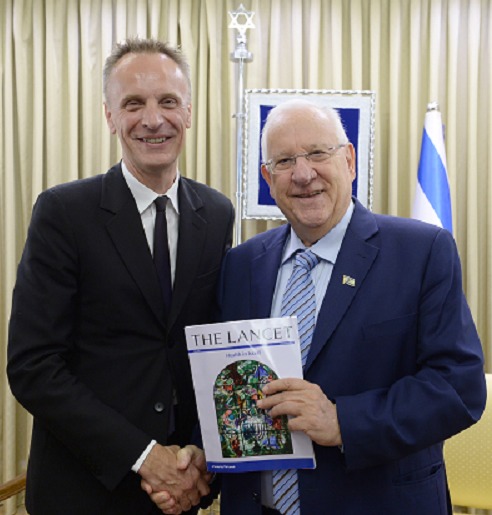
Professor Richard Horton, Editor-in-Chief of *The Lancet* with Israeli President Reuven Rivlin. Professor Richard Horton (left) and President Reuven Rivlin (right) with a copy of the special issue of *The Lancet* dedicated to Israel’s healthcare system and medical innovations in Israel. (Photo: Mark Neiman / GPO).

Two-and-a-half years later, during May 8–11, 2017, special events were held in Tel-Aviv, Haifa, Nazareth, Beer-Sheva, and in Jerusalem (at the residence of the President of Israel, Figure 1) to launch the publication of a series in *The Lancet* entitled “Health in Israel.”^6^ The issue comprises 12 articles, essays, or commentaries in total, as well as online-only profiles—all written by or about Israeli health professionals and researchers, regarding a variety of aspects of medicine and health care in Israel. At a press conference in Tel Aviv, Horton concluded by saying: “The special issue on Israel will not be a one-time project. It is the beginning of a close partnership.”

You can access all of these articles—free—at http://www.thelancet.com/series/health-in-israel or in the online June 24, 2017 issue of *The Lancet*.^6^

What lessons have we learned from this story?

We have learned that a scientific medical journal can provide a political perspective regarding issues that impact the health of a nation.

We have learned that if a journal makes the decision to support a particular political perspective, they must first conduct the necessary research to clarify the facts. This was the ancient wisdom imparted by Aesop (620–564 BC) in his fable “The Mule:” there are two sides to every story—and then there’s the truth. While research may not provide the absolute “truth,” it is critical to make strides in the right direction by providing an opportunity for response from the “other side,” before drawing conclusions or taking definitive action. This is also the lesson of the Talmudic wisdom that usually favored the opinions of the Academy of Hillel over those of the Academy of Shammai—simply because the former always took the time to quote the latter. This is also an approach which welcomes disagreement, so long as disagreement is followed by dialogue attempting to seek the truth (even when elusive) rather than disagreement for the sake of destructive and self-serving conflict.

Finally, we can see, beyond the shadow of a doubt, that boycotts are a double-edged sword harming not only those who are boycotted, but the boycotters themselves. An academic boycott ultimately prevents the very process that can create positive change—cordial academic discourse. I have used this term several times, yet I cannot stress it enough. “Cordial” is that which treats the other with respect—in the midst of disagreement; “academic” relates to research, facts, and proper handling of such; and “discourse” relates to the orderly interchange (i.e. bi-directional) of ideas. Any academic boycott, declared or secretive, undermines academia by its refusal to respect the other side; its refusal to research the facts from all possible angles; and its refusal to enter into an exchange of ideas. Only dialogue and discussion with mutual respect can facilitate reconciliation, bring opposing parties closer together, and even solve challenging problems.

It is possible that the future will see differing and even contentious points of view and opinions regarding policies that affect matters of health and social well-being on the pages of *The Lancet*, *Rambam Maimonides Medical Journal*, or in other academic venues. We have learned that so long as expression of such differences and disagreements is conveyed in a manner that reflects integrity, respects dignity, avoids generalizations, and shuns vituperative innuendo, thereby allowing for continued constructive discussion in a joint search for truth—then such expression can and must be encouraged as a healthy part of academic discourse.

In conclusion, there is a saying, “there is none so blind as those who will not see.” Let the nay-sayers continue to say nay. This cannot stop the progress of cordial academic discourse for those who are bold enough to go against the flow for the benefit of humankind. I am proud to be part of a tradition that seeks for open communication and discussion in order to facilitate reconciliation.
